# Inhibitors of MAO-A and MAO-B in Psychiatry and Neurology

**DOI:** 10.3389/fphar.2016.00340

**Published:** 2016-10-18

**Authors:** John P. M. Finberg, Jose M. Rabey

**Affiliations:** ^1^Rappaport Faculty of Medicine, Technion, Israel Institute of TechnologyHaifa, Israel; ^2^Assaf Harofe Medical Center, Affiliated to Sackler School of Medicine, Tel Aviv UniversityTel Aviv, Israel

**Keywords:** rasagiline, selegiline, safinamide, depression, Parkinson's disease

## Abstract

Inhibitors of MAO-A and MAO-B are in clinical use for the treatment of psychiatric and neurological disorders respectively. Elucidation of the molecular structure of the active sites of the enzymes has enabled a precise determination of the way in which substrates and inhibitor molecules are metabolized, or inhibit metabolism of substrates, respectively. Despite the knowledge of the strong antidepressant efficacy of irreversible MAO inhibitors, their clinical use has been limited by their side effect of potentiation of the cardiovascular effects of dietary amines (“cheese effect”). A number of reversible MAO-A inhibitors which are devoid of cheese effect have been described in the literature, but only one, moclobemide, is currently in clinical use. The irreversible inhibitors of MAO-B, selegiline and rasagiline, are used clinically in treatment of Parkinson's disease, and a recently introduced reversible MAO-B inhibitor, safinamide, has also been found efficacious. Modification of the pharmacokinetic characteristics of selegiline by transdermal administration has led to the development of a new drug form for treatment of depression. The clinical potential of MAO inhibitors together with detailed knowledge of the enzyme's binding site structure should lead to future developments with these drugs.

## Introduction

Monoamine oxidase (MAO; EC 1.4.3.4.) is a widely distributed mitochondrial enzyme with high expression levels in gastro-intestinal and hepatic as well as neuronal tissues. The enzyme catalyzes the oxidative deamination of a variety of monoamines, both endogenous and exogenous, and has major roles in metabolizing released neurotransmitters, and in detoxification of a large variety of endogenous and exogenous amines. Drugs which inhibit MAO are currently in clinical use for treatment of affective disorders and Parkinson's disease (PD). In this chapter we review recent developments in the basic pharmacology of MAO inhibitors (MAOI) and their clinical usage, and discuss the potential for new drug development in this field.

The overall enzyme reaction of MAO can be represented by the following equation:

$$\begin{array}{l} \begin{array}{c} \text{ MAO}\\ {\text{R}-\text{CH}}_{2}-{\text{NH}}_{2}+{\text{O}}_{2}+{\text{H}}_{2}\text{O}=\text{R}-\text{CHO}+{\text{NH}}_{3}+{\text{H}}_{2}O_{2} \end{array} \end{array}$$

The aldehydes produced by the action of MAO are metabolized further by aldehyde dehydrogenase and aldehyde reductase leading to the formation of glycols and carboxylic acids (Westfall and Westfall, 2011). The fact that an aldehyde is formed initially together with H_2_O_2_ which can generate reactive oxygen species (ROS) has drawn attention to the possibility that products of the action of MAO may be neurotoxic (Jenner, 2003). In this connection it should be realized that ROS and other reactive species are normally metabolized by scavenger enzymes including catalase and superoxide dismutase, and dysfunction of these enzyme systems may be a factor in neurodegenerative disease (Aluf et al., 2011). The dopaminergic neurons of substantia nigra pars compacta (SNpc) are at risk to oxidative stress because of their tonic activity and dense packing. Their degree of oxidative stress increases in early PD when a portion of the neurons have been lost, and the activity of the remaining ones increases in compensation. This situation was modeled recently in a microdialysis study in which a non-diffusible indicator molecule was perfused through a probe placed in the striatum. Following intraventricular injection of 6-hydroxydopamine in a dose adequate to reduce SNpc dopaminergic cell number by 50%, the level of oxidative stress increased markedly (Aluf et al., 2011), and was reduced following systemic injection of an MAO-A or MAO-B inhibitor (Aluf et al., 2013).

## MAO isoforms

Two isoenzymes are encoded in the human X-chromosomal gene Xp1 123, MAO-A, and MAO-B. The two forms have over 70% homogeneity. Biochemically, the two forms can be differentiated by their substrate and inhibitor specificities; MAO-A shows greater affinity for hydroxylated amines such as noradrenaline (NA) and serotonin (5-hydroxytryptamine, 5-HT), whereas MAO-B shows greater affinity for non-hydroxylated amines such as benzylamine and beta-phenylethylamine (PEA). The amines dopamine (DA) and tyramine show similar affinity for each enzyme form. Clorgyline is a selective inhibitor of MAO-A while selegiline (*l*-deprenyl) and rasagiline are relatively selective inhibitors of MAO-B. The ratio of selectivity of selegiline and rasagiline for MAO-B is such that in human subjects, doses 2–5 fold higher respectively than the MAO-B selective dose can cause significant inhibition of MAO-A as shown by tyramine pressor responses (Bieck and Antonin, 1994; Goren et al., 2010). Some inhibitors can inhibit both forms of the enzyme (referred to as non-selective inhibitors, although this can cause confusion because the inhibitors are quite selective for MAO as opposed to other enzymes). The precise localization of the two MAO isoforms in brain has not been completely elucidated. Studies using cell cultures (Yu and Hertz, 1983; Carlo et al., 1996) pointed to localization of MAO-A within glial cells, but this is not true for the intact brain, where investigations in both primate and non-primate species have established that the glial enzyme is predominantly type B (Levitt et al., 1982; Denney and Denney, 1985; Westlund et al., 1988). The type A isoform has been localized to several neuronal cell types in primate and rodent species, including NA-ergic neurons of the locus coeruleus, DA-ergic neurons of the substantia nigra pars compacta (SNpc), (Westlund et al., 1993) and striatal medium spiny neurons (Sader-Mazbar et al., 2013). Serotonergic neuronal cell bodies of the raphe nucleus stain positive for MAO-B but the isoform localized to axonal varicosities may be of the A isoform (Denney and Denney, 1985). Selective inhibition of MAO-A leads to increased levels of neurotransmitter within noradrenergic (NA-ergic) and 5-HT-ergic neurons of the CNS, and clinical antidepressant action, while inhibition of MAO-B leads to increased levels of DA in the Parkinsonian brain with partial depletion of DA-ergic neurons in SNpc, and anti-Parkinsonian action (see Finberg, 2014 for a detailed description of these events at the synaptic level).

Many compounds with MAO inhibitory properties are being prepared by researchers, however the present account is limited to a description of the most important drugs from a therapeutic viewpoint, i.e., affective disorders and Parkinson's disease. The chemical structures of drugs mentioned in this review and a brief description of their major characteristics is shown in Table 1.

**Table 1:** **Structures and major characteristics of MAO inhibitors mentioned in the text**.

**Compound**	**Activity**	**Status**	**Chemical structure**
Phenelzine	Irreversible MAO-A + MAO-B	Used as antidepressant Hepatotoxicity Needs dietary control for restriction of tyramine intake	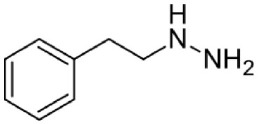
Tranylcypromine	Irreversible MAO-A + MAO-B	Used as antidepressant with dietary control	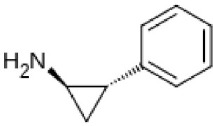
Pargyline	Irreversible MAO-A and MAO-B	Antidepressant and antihypertensive Currently not in clinical use	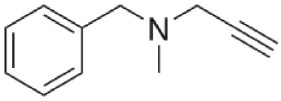
Selegiline	Irreversible MAO-B selective (R- enantiomer) Selectivity is dose dependent *in vivo*	Metabolism to amphetamines	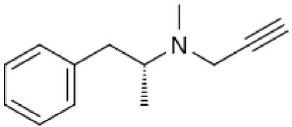
Clorgyline	Irreversible highly MAO-A selective	Antidepressant effect demonstrated in humans but not in clinical use	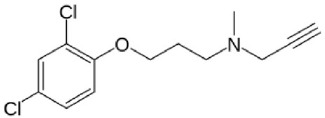
Moclobemide	Reversible highly MAO-A selective	Moderately effective antidepressant drug	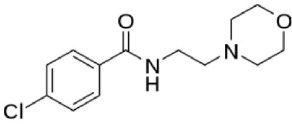
Rasagiline	Irreversible MAO-B selective (R+ enantiomer) Selectivity is dose dependent *in vivo*	Neuroprotective *in vitro*, anti-Parkinson drug, metabolism to 1-aminoindan	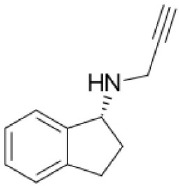
Safinamide	Reversible highly MAO-B selective	Anti-Parkinson drug, glutamate receptor antagonistic and Na+ channel blocking properties	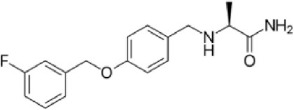
Ladostigil	MAO-A + MAO-B Relative brain selectivity, minimal tyramine potentiation	Cholinesterase and MAO inhibition	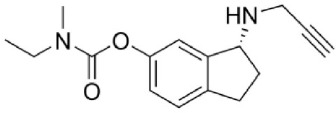
VAR 10303	MAO-A + MAO-B Relative brain selectivity, minimal tyramine potentiation	Fe chelation and MAO inhibition	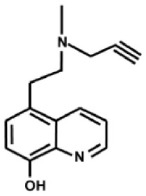
M30	MAO-A and MAO-B Relative brain selectivity	Fe chelation and MAO inhibition	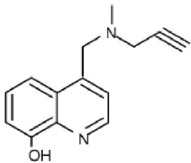

## Molecular structure of MAO and mechanism of enzyme inhibition

For many years, a formula was searched for without success to explain the selectivity of an inhibitor molecule for MAO-A or MAO-B. The problem was solved when the MAO protein molecule was crystallized by the groups of Edmondson and Sukihara, enabling three-dimensional modeling of the protein and its combining site (Binda et al., 2002, 2007; De Colibus et al., 2005; Son et al., 2008). It was then seen that a two-site cavity structure exists for human MAO-B (hMAO-B), with an entry cavity and a reactive site cavity, whereas in human MAO-A (hMAO-A) the active site cavity is not bipartite and is shorter and wider than the longer and narrower substrate cavity in hMAO-B (De Colibus et al., 2005). The reactive site contains a combining moiety in which the N5 atom of FAD is displayed on the inner surface, and tyrosines 398, and 435 guard the entry gate in hMAO-B (Binda et al., 2002). Knowledge of the three-dimensional aspects of these sites, and the associated amino acid positions, can now be utilized in the design of new inhibitors.

Both reversible and irreversible inhibitors of MAO have been developed in previous years, and are currently in use clinically for treatment of affective and neurological disorders. Irreversible inhibitors are of several types: hydrazines, cyclopropylamines, and propargylamines. In all cases, these drugs combine covalently with the N5 atom of the flavin residue, but the rate of dissociation of the drug-enzyme complex is variable. Detailed mechanisms for the drug-enzyme complex formation have been described. Following recognition of the enzyme pharmacophore by the drug, the inhibitor molecule is metabolized leading to a reactive intermediate which combines covalently with the N5 atom of FAD leading to formation of a drug-receptor adduct, which then undergoes aging and irreversible combination. The term “suicide inhibitor” has been used in description of this type of drug action (see Finberg, 2014).

In general, because these inhibitors will irreversibly inactivate the enzyme, their action can only be reversed by generation of new enzyme molecules, a process which can take days or weeks. In clinical use, the drugs are administered daily, using a dose which alone is adequate to cause only partial enzyme inhibition, but when given daily over several days will cause a cumulative inhibition up to 90% or more of the target enzyme in brain. Continued drug administration ensures that newly-formed enzyme molecules are also inhibited, and that the enzyme activity is maintained at a constant low level. The clinical importance of this type of drug use is that a constant high degree of enzyme inhibition can be maintained over time. In addition, however, on stopping treatment enzyme activity will remain at a low level even after the drug itself has been cleared from the body.

## Treatment of depression with MAO inhibitors

### Non-subtype-selective irreversible inhibitors

The profound antidepressant action of MAOI inhibitors was discovered by chance (Lehmann and Kline, 1983) in tuberculous patients treated with iproniazid, a derivative of the hydrazine compound isoniazid. Further developments led to the introduction into clinical use of several non-subtype-selective irreversible MAO inhibitors including the hydrazines phenelzine and isocarboxazid, the propargylamine pargyline, and the cyclopropylamine tranylcypromine, but these compounds can all lead to potentiation of the cardiovascular effects of the dietary amine tyramine (“cheese effect”). Following realization that the cheese effect can be avoided by dietary counseling, and that MAO inhibitors are in fact excellent drugs for treatment of drug-resistant and atypical depression, use of certain non-subtype-selective inhibitors, in particular tranylcypromine (Parnate), is now seen with increasing frequency. Tranylcypromine has pharmacological properties in addition to inhibition of MAO, in particular inhibition of lysine-specific histone demethylation type 1, and interaction with the endogenous cannabinoid system (Lee et al., 2006; Hill et al., 2008). Phenelzine also has an additional pharmacological property which may be involved in its antidepressant actions, namely blockade of GABA and alanine transaminases (Baker et al., 1991; Todd and Baker, 2008).

In a recent review (Heijnen et al., 2015) of a small number of clinical trials, tranylcypromine was found to be an efficacious and safe drug for the treatment of bipolar depression, when administered with correct dietary counseling. Although cheese effect is a potentially serious reaction, the limitations it imposes on treatment of psychiatric patients have been much exaggerated, because the amounts of tyramine occurring in foodstuffs are quite low, and only a gross violation of normal dietary directions would be likely to cause a fatal, or damaging, reaction (Gillman, 2011). The management of such a hypertensive reaction if it does occur has also been well-documented (Gillman, 2011). In addition to cheese effect, another potential danger is serotonin toxicity syndrome (ST), which can occur following the combination of irreversible MAOI with a drug which has the potential to elevate 5-HT synaptic levels, such as a serotonin-selective reuptake inhibitor (SSRI) (Gillman, 2006). In this context, the relatively long period required for return of MAO activity following cessation of therapy with an irreversible inhibitor is important when a change in therapy is required. If therapy with a SSRI is to be used, there is a danger of ST if these drugs are instituted before MAO activity has returned to normal levels. Following cessation of tranylcypromine administration in healthy subjects, a period of 30 days was required for complete normalization of the pressor response to oral tyramine challenge (Bieck and Antonin, 1988). In the case of rasagiline, using platelet activity of MAO-B as the index, enzyme activity returned to baseline levels 2 weeks after cessation of drug administration in healthy subjects (Thebault et al., 2004). The time required for return of enzyme activity in the brain however, is considerably longer than in the periphery. Using (Denney and Denney, 1985) C-labeled selegiline together with positron emission tomography (PET) the half-time for return of MAO-B binding in the brain following complete blockade of binding by an initial injection of selegiline in a baboon was 30 days (Arnett et al., 1987), and using similar technique, following initial MAO-B inactivation by rasagiline in human subjects, was 40 days (Freedman et al., 2005). Recommended periods (by manufacturer) for wash-out after cessation of tranylcypromine range from 7 to 10 days (Gahr et al., 2013).

The antidepressant effect of MAOI has focused interest on the possibility that altered expression levels of the MAO enzyme could be the cause of some forms of depressive disorders. Polymorphisms in the MAO-A gene have been associated with a number of behavioral traits. Reduced enzyme activity is associated with violent behavior and aggression, whereas over-expression may be linked to depression (Alia-Klein et al., 2008). These facts, together with the well-known biogenic amines hypothesis, provide theoretical background in support of the use of MAOI for treatment of affective disorders. Several studies have attempted to link the MAOA-uVNTR polymorphism, which leads to increased enzyme transcription, with suicidal tendency, but a meta-analysis including 1452 psychiatric patients and 1198 control subjects did not find a significant association (Hung et al., 2012) with this particular trait. In a recent study in which MAO-A expression level (total distribution volume, Vt, of ^11^C-harmine by PET) was determined in borderline personality disorder (BPD) patients, the MAO-A brain content was correlated with symptom severity (Kolla et al., 2015). Interestingly, MAO-A Vt was increased in prefrontal cortex and anterior cingulate cortex by 43 and 42% respectively in severe BPD subjects in relation to controls.

One of the main restrictions to the clinical use of MAOI for treatment of depression is the cheese effect. In preclinical and clinical studies it was shown that potentiation of the pharmacological effects of tyramine occurs following selective inhibition of MAO-A but not MAO-B (Lader et al., 1970; Finberg and Tenne, 1982; Finberg and Gillman, 2011). This can be attributed to the localization of MAO-A to noradrenergic (as well as serotonergic) neurons (see Finberg, 2014) for detailed review). A corollary to this selective localization of MAO subtypes is that selective inhibitors of MAO-A but not MAO-B are effective antidepressants (Youdim and Finberg, 1983), however no irreversible selective MAO-A inhibitors are in use for treatment of depression.

### Reversible inhibitors of MAO-A

In the 1980s several groups of researchers prepared selective reversible inhibitors of MAO-A (RIMAs) (Tipton et al., 1984) based on the theory that if substrate levels increased as a result of inhibition of the enzyme, the degree of enzyme inhibition would be reversed by increased dissociation of the inhibitor from its combining site, i.e., a reversible inhibitor would possess a built-in safety factor in the case of tyramine ingestion, and therefore reversible inhibitors would not cause cheese effect (Finberg, 2014). The correctness of this notion was confirmed in a number of clinical studies using tyramine challenges in patients (Finberg and Gillman, 2011; Finberg, 2014). Currently, moclobemide is the only RIMA available for clinical use. Although clinical studies carried out in the period following its general release showed an efficacy similar to that of tricyclic antidepressants (TCA) for treatment of depression it was found less effective than irreversible MAOIs (Lotufo-Neto et al., 1999; Shulman et al., 2013). Another reversible selective MAO-A inhibitor with antidepressant properties is methylene blue (Naylor et al., 1987; Ramsay et al., 2007). This interesting drug has several pharmacological actions, including inhibition of nitric oxidase synthase (NOS), and guanylate cyclase (Harvey et al., 2010), and so its antidepressant properties should not be solely ascribed to inhibition of MAO-A.

### Selective inhibitors of MAO-B in treatment of depression and attention deficit hyperactivity disorder (ADHD)

Following introduction of the irreversible selective MAO-B inhibitor selegiline for treatment of PD (see following sections), its efficacy for treatment of depression was examined in several uncontrolled clinical trials, using the MAO-B-selective dose of 10 mg daily, and as was anticipated following the known involvement of mainly serotonergic and noradrenergic neuronal systems in depression, it was not effective. When examined at the higher doses of 30 or 60 mg daily, however, it did have significant antidepressant effect, especially in treatment-resistant depression (Mann et al., 1989; Sunderland et al., 1994). Based on these positive results a pharmacokinetic strategy was developed (selegiline transdermal system, STS) which permits a greater portion of the administered dose to enter the CNS, and reach tissue levels concomitant with inhibition of both MAO-A and MAO-B while avoiding inactivation of gastro-intestinal and hepatic enzyme (Mawhinney et al., 2003). This technique was developed on the basis of preclinical experiments in guinea-pigs (Mawhinney et al., 2003). In order to understand the mechanism of this relative brain selectivity, it is necessary to understand: (a) that selegiline is only MAO-B selective at low dose, and higher doses will inhibit both MAO-A and MAO-B, and (b) the pharmacokinetics of selegiline (Magyar, 2011). This compound is based on the molecule of R(−)methamphetamine, and following systemic administration it is metabolized by cytochrome P450 enzymes in the liver, mainly to R(−)methamphetamine, R(−)amphetamine, and N-desmethylselegiline (Laine et al., 2000; Azzaro et al., 2007). When administered transdermally the first-pass metabolism is largely avoided, and a larger part of the administered dose directly accesses the brain, and binds irreversibly to both MAO-A and MAO-B. Intact molecules of the drug which leave the brain will be metabolized in the liver, but large scale inhibition of MAO isoforms in gastro-intestinal tract and liver will be avoided (Mawhinney et al., 2003), and the formation of potentially damaging amphetamines is reduced. The success of this strategy has been confirmed in human experiments, in which it was shown: a) that STS is an effective antidepressant, and b) that at antidepressant doses it does not cause cheese effect (Azzaro et al., 2006; Blob et al., 2007). Another dose form of selegiline aimed to produce a similar alteration in pharmacokinetics of the drug is the buccally administered solution (Zydis selegiline) which similarly produces effective antidepressant activity without significant tyramine potentiation (Clarke et al., 2003a,b). Its improved pharmacokinetics permit the use of lower doses which confer greater selectivity for MAO-B over MAO-A inhibition. The MAO-B-selective inhibitor rasagiline has been found effective in treatment of PD depression with a greater response at 2 mg/day than the usual dose of 1 mg/day, possibly because of the greater inhibitory effect on MAO-A at the higher dose (Korchounov et al., 2012).

In preclinical studies in rats, rasagiline administered at selective MAO-B-inhibitory dose did not modify DA, NA or 5-HT levels or induce reserpine reversal (Finberg and Youdim, 2002), however in aged mice, chronic administration of an MAO-B-selective dose (0.2 mg/kg daily for 3 weeks) did increase brain levels of DA and reduce DOPAC, and also showed antidepressant-like effects. Interestingly, the drug returned activity in behavioral paradigms such as learning and forced-swim test, which were reduced in the aged animals, to levels seen in young animals (Weinreb et al., 2015). It is of interest that these effects of rasagiline in aged mice were produced also by chronic administration of its major metabolite 1-aminoindan, at a dose of 5 mg/kg daily over 3 months (Badinter et al., 2015). The authors of these articles suggested that the effects of 1-aminoindan indicate an action on catecholaminergic systems which is not the result of MAO inhibition, because *ex vivo* brain MAO activity was not inhibited; however since it is a reversible MAO inhibitor (Binda et al., 2005) their *ex vivo* assay of MAO would not be expected to show a change in enzyme activity, because the drug would be washed out or diluted in the brain homogenate used in their assay. On the other hand, the changes in tissue monoamine levels and their metabolites (Mann et al., 1989) are indicative of an inhibition of MAO.

In a placebo-controlled study of 11 children with ADHD selegiline significantly improved attention but not impulsivity (Akhondzadeh et al., 2003; Niederhofer, 2003; Rubinstein et al., 2006). In three studies in which selegiline was compared with methylphenidate in children with ADHD, the two drugs had similar efficacy (Akhondzadeh et al., 2003; Niederhofer, 2003; Mohammadi et al., 2004). Considering the detrimental pharmacology of amphetamine-like drugs used in ADHD, the use of MAO-B inhibitors in this condition warrants further study. Since MAO-B inhibition markedly increases the brain levels of endogenous PEA (“the body's own amphetamine”), this could be an explanation for the selegiline effects observed in the above studies, in addition to inhibition of DA breakdown.

### MAOI and drug addiction

Use of MAOI in treatment of depression in cocaine-addicted subjects has been proposed, because chronic cocaine administration reduces the activity of monoamine neurotransmitter systems, which are enhanced by MAOI. In addition, by enhancing DA levels MAOI could possibly be used to substitute for the reward-initiating effect of cocaine (Ho et al., 2009). The potential of MAOI to reduce cocaine-induced reward was studied in mice (Ho et al., 2009). The long-term administration of both selegiline (1 mg/kg i.p. daily for 3 weeks) and pargyline (10 mg/kg i.p. daily for 3 weeks) abolished cocaine-supported operant responses whereas long-term treatment with clorgyline (2 mg/kg i.p. daily for 3 weeks) did not. It should be noted that the doses of selegiline and pargyline used were probably adequate to inhibit both MAO-A and MAO-B, as shown by their reduction of dihydroxyphenylacetic acid (DOPAC) and 5-hydroxyindole acetic acid (5-HIAA) levels in frontal cortex, while clorgyline enhanced 5-HT but did not reduce 5-HIAA levels in frontal cortex. The authors proposed that the use of MAO-B inhibitors to curb cocaine reward should be further considered. In a pilot study in human subjects, 10 mg p.o. selegiline daily reduced cocaine consumption but in a subsequent larger study (300 subjects) transdermal selegiline did not significantly reduce consumption. An additional study with transdermal selegiline reduced cocaine-related scores of anger and tension as well as craving but also did not reduce subjective reported rewarding effects of a higher dose of cocaine compatible with binge use in humans (Elkashef et al., 2006; Harris et al., 2009). Clinical studies have not found evidence of abuse liability in humans (Yasar et al., 1996), and in addition it should be noted that selegiline does not induce addictive behavior in monkeys (Winger et al., 1994).

### Pharmaco-therapy of PD depression

There are a number of considerations relating to the pharmaco-therapy of PD depression, including the stage of the disease, possible interactions with other medications (especially L-dopa, LD), control of the autonomic nervous system, and the disturbed normal balance between the monoamine systems of the brain. In addition, the possibility of cognitive deficits and PD dementia will confuse the understanding of the patient's affective state. In a meta-analysis of 11 controlled clinical trials for pharmacological treatments in PD depression between the years 2004 and 2014 (Sandoval-Rincon et al., 2015), rasagiline was found effective, but at the dose of 2 mg/day, which is higher than the usual dose of 1 mg daily for PD symptomatology.

For advice to clinicians on the ins and outs of treatment of depression with MAOIs, the reader is referred to recent reviews (Cohen and Sclar, 2012; Goldberg and Thase, 2013; Shulman et al., 2013).

## Neuroprotective actions of MAOI

All MAOI possess inherent neuroprotective properties because of their inhibition of H_2_O_2_ and toxic aldehyde release following oxidative metabolism of amines, however individual inhibitors may possess an intrinsic neuroprotective action in addition. Selegiline was found by Knoll and co-workers to increase the natural life-span of laboratory rats, and subsequently was found to exert an anti-apoptotic effect in a variety of tissues and cells which was independent of MAO inhibition (Tatton and Chalmers-Redman, 1996). It is important to note that the anti-apoptotic properties of desmethylselegiline (the selegiline metabolite which is the active neuroprotective molecule) are superior to those of selegiline, and that R(−)methamphetamine, the major metabolite of selegiline, antagonizes the neuroprotective property of selegiline and desmethylselegiline (Tatton and Chalmers-Redman, 1996). Subsequently, rasagiline was found to also possess neuroprotective properties both *in vivo* (Aluf et al., 2013) and *in vitro* (Finberg et al., 1998; Weinreb et al., 2011). Both these molecules increase BCl_2_ and PKC-epsilon levels, enhance synthesis and release of BDNF and GDNF, and activate additional anti-apoptotic mechanisms (Jenner and Langston, 2011). It is a current unsolved mystery why these small molecules should exert these complex pro-survival effects. One series of studies produced evidence that selegiline binds to GAPDH and prevents the nuclear translocation of this enzyme (Carlile et al., 2000), however the tricyclic selegiline derivative CGP3466 (Omigapil), which does not inhibit MAO, binds GAPDH and prevents its nuclear translocation, possesses anti-apoptotic activity *in vitro* and *in vivo* (Waldmeier et al., 2000) but was not effective in clinical trials for PD and ALS. Rasagiline also prevents the pro-apoptotic nuclear translocation of GAPDH (Maruyama et al., 2001). The rasagiline metabolite 1(R)-aminoindan possesses anti-apoptotic activity (at higher concentrations than the parent molecule), and shows a similar spectrum of biochemical mechanisms as described for rasagiline (Bar-Am et al., 2010), however the presence of the propargyl moiety seems to be an important factor in neuroprotection, since propargylamine itself also possesses anti-apoptotic activity, albeit at higher concentrations than are needed with rasagiline or selegiline (Weinreb et al., 2005).

### Compound molecules with MAO inhibitory and neuroprotective properties

Ladostigil (Weinstock et al., 2000) is a compound molecule consisting of a molecule of rasagiline with the addition of a propylcarbamate moiety, which confers cholinesterase-inhibiting properties. The combination of these two moieties in the same molecule produced a drug with inhibitory properties on both enzymes *in vivo*, while it is ineffective *in vitro*. An additional fortuitous aspect of this molecule is that its MAO-inhibitory property is brain-selective, so that the likelihood of cheese effect is small. In rats, an oral dose of 75 μmoles/kg daily for 2 weeks inhibits brain cholinesterase by 40% and brain MAO-A and –B by 70% with no significant inhibition of intestinal or hepatic MAO (Weinstock et al., 2000), while higher doses can produce nearly complete inhibition of brain MAO in several species (Youdim et al., 2005).

The active metabolite of ladostigil responsible for the cholinesterase-inhibitory activity is R-MCPAI (6-(N-methyl carbamyloxy)-1(R)-aminoindan hydrochloride), which is formed from ladostigil by CYP-2C19, while MAO inhibitory activity *in vivo* is due to the metabolite R-HPAI (6-hydroxy-N-propargyl-1(R)-aminoindan mesylate), since ladostigil itself does not inhibit MAO *in vitro*. Recently the self-limiting inhibitory effect of ladostigil on cholinesterases (maximal inhibition level is 50–55%) has been studied in mice, and found to be the result of rapid hydrolysis of the complex between R-MCPAI and the enzyme, not by limitation in formation of R-MCPAI (Moradov et al., 2015). A slow rate of conversion of ladostigil to R-HPAI in the intestine prevents significant inhibition of intestinal MAO, although following absorption of the parent molecule it is converted to R-HPAI in the brain but to a lesser extent in other tissues.

The drug was also found to possess a variety of neuroprotective and cognitive effects in animal models (Weinreb et al., 2012). Mechanistic studies showed that ladostigil binds to the VDAC mitochondrial complex, protecting against reduction in mitochondrial potential, and activates alpha-secretase leading to production of the non-amyloidogenic form of soluble APP by a MAP-Kinase dependent mechanism, in a similar way to rasagiline (Yogev-Falach et al., 2002). Ladostigil possesses anti-apoptotic properties against apoptosis induced by the naturally-occurring neurotoxin N-methyl(R)salsolinol and the peroxynitrite-generating molecule SIN-1 in SH-SY5Y cells (Maruyama et al., 2003). It also possesses anti-inflammatory properties as shown by its ability to reduce TNFα levels in mouse spleen and macrophages, following LPS stimulation (Moradov et al., 2015) and reduces the extent of gliosis and memory deficits following streptozotocin-induced lesions of the CNS in rats (Shoham et al., 2007). Ladostigil was tested in old rhesus monkeys for cognitive behavioral effects, and was found to improve attention (Buccafusco et al., 2003). Administered to stressed pregnant rats it corrected the depressive behavior of the male offspring (Goelman et al., 2014). The drug is currently in clinical trial for Mild Cognitive Impairment (MCI).

Additional multi-target inhibitors of MAO have been developed with the aim of incorporating iron-chelating activity together with MAO inhibition (Wang et al., 2014). M30 and VAR10303 are both in development for clinical use in neurodegenerative diseases. These compounds are relatively selective inhibitors of brain as opposed to intestinal and liver MAO, with similar degree of inhibitory activity on MAO-A and MAO-B (Gal et al., 2005; Bar-Am et al., 2015). Unlike ladostigil, M30 (Zheng et al., 2005) has potent MAO inhibitory activity *in vitro*. The reason for the brain selectivity of these compounds has not yet been determined.

## Antiparkinsonian features of MAO-B inhibitors

Parkinson's disease (PD) is a neurodegenerative disorder primarily of the nigrostriatal DA-ergic pathway that affects the motor system and results in symptoms including uncontrollable tremor, muscle rigidity, slowness of movement (bradykinesia) and postural instability (Lang and Lozano, 1998a). It is estimated that it affects more than 4 million people worldwide (Schapira et al., 2005), significantly shortens life, and affects quality of life (Lang and Lozano, 1998a,b).

Much effort is being directed to development of new drugs with neuroprotective properties for the treatment of neurodegenerative diseases. Prior to initiating a new drug development, evidence of target engagement is desirable. In the case of putative neuroprotective therapies for PD this is not readily accomplished. There are few targets in the CNS that are associated with a possible pathogenic mechanism and are accessible to drug treatment, although as described above, MAOI have potential neuroprotective properties. Another problem is that existing assessment scales have a limited range and are particularly insensitive to detecting the modest change in movement that occurs in the early stages of the disease. Identification of a validated biomarker would be a significant advance in diagnosis and objective measurement of disease progression and drug efficacy. Recently Moloshnikov and coworkers (Molochnikov et al., 2012) reported a disease signature using blood RNA that detects idiopathic PD with a sensitivity of 90%. This may help to improve the selection of PD patients for clinical trials. The population of PD patients selected for inclusion in clinical trials is also critically important. Patients in early disease stages are frequently selected because they are likely to have a larger number of remaining neurons that can potentially be protected or rescued than patients in advanced stages of the disease (Fearnley and Lees, 1991). In addition, patients in early stages of the disease are generally still untreated which avoids the complication of confounding drug action. There is, however, a greater possibility of inaccurate diagnosis in early disease stage and a higher risk of dropout if patients require treatment. Moreover, disease progression is slower in the early stages of the disease, possibly as a result of more efficient functional compensation (Rascol et al., 2011).

Symptoms of PD are improved with DA–replacement therapies such as DA receptor agonists and LD. Over time, however, the benefit of these drugs fluctuates and patients begin to experience loss of benefit with each dose of LD (wearing–off) and involuntary dyskinesia. In addition some parkinsonian symptoms including disturbances of gait and tremor may be resistant to DA-ergic therapy.

The way in which selective inhibition of MAO-A or MAO-B modifies DA release *in vivo* was studied in the rat by microdialysis. Initial studies were made by single dose administration using non-selective doses of the MAOI. The first study to employ MAO subtype-selective doses of clorgyline, selegiline, and rasagiline given chronically was carried out by Lamensdorf and colleagues (Lamensdorf et al., 1996). This study showed clearly that all three MAOI could increase striatal extracellular levels of DA when given over 3 weeks, although clorgyline caused the greatest elevation in DA levels. In a later study by the same group (Lamensdorf et al., 1999), a differential effect of selegiline was found to increase expression of the DA transporter (DAT), whereas rasagiline and clorgyline did not. In a follow-up study (Sader-Mazbar et al., 2013), the effect of rasagiline and clorgyline on LD-induced DA levels was studied in rats with a unilateral 6-hydroxydopamine (6-OHDA) lesion of the substantia nigra. This study showed clearly that clorgyline again was the most effective MAOI in causing an increase in DA extracellular levels, although rasagiline also elevated DA levels, in a dose level which was selective for MAO-B inhibition. The superior effect of clorgyline in elevating striatal extracellular DA levels is thought to be the result of inhibition of the neuronal MAO, whereas the effectiveness of MAO-B inhibitors in enhancement of LD-induced DA output in the brain with a lesion of the DA-ergic neurons of substantia nigra is thought to be the result of inhibition of glial cell MAO-B (Carlo et al., 1996).

Inhibition of MAO-B may conserve the depleted synaptic levels of DA, and delay the need for treatment with LD in patients with early-stage PD. In patients with advanced-stage PD who experience fluctuations in response to LD, MAO-B inhibition potentiates and prolongs the effect of LD and permits use of a lower dose (Riederer and Laux, 2011).

Irreversible MAOI selective for type B of the enzyme are among the earliest drugs used in PD. They can be used with or without LD (Riederer and Laux, 2011). Both selegiline and rasagiline are beneficial in treating motor symptoms in PD as monotherapy as well as in combination with LD and a decarboxylase inhibitor. The main differences between the two drugs are related to their metabolism, interaction with cytochrome P450 enzymes and quantitative properties at the molecular biologic/genetic level. Rasagiline is more potent as shown in the daily dose necessary for a symptomatic effect: selegiline 5–10 mg daily and rasagiline 1 mg daily.

### Selegiline

Selegiline is a useful treatment for PD symptoms both in monotherapy and as adjunct therapy to LD (Riederer et al., 2004). However, selegiline undergoes first-pass metabolism to R(−)amphetamine and R(−)methamphetamine, which have the potential to cause cardiovascular and CNS adverse effects (Gal et al., 2005). The contribution of these metabolites to selegiline's clinical symptomatic effects (Elsworth et al., 1982) and the possibility of adverse cardiovascular reactions (Churchyard et al., 1997), has often been discussed. R(−)amphetamine has about one-tenth the activity of S(+)-amphetamine on the sympathetic nervous system but the enantiomers are equivalent in antagonism of DA uptake in the striatum (Coyle and Snyder, 1969). One clinical study in which the effects of 10 mg of selegiline were compared with equivalent doses of R(−)-amphetamine and R(−)-methamphetamine concluded that only selegiline, and not its metabolites, possesses antiakinetic efficacy in Parkinsonian patients (Elsworth et al., 1982). Moreover, from documentation of side effects in the large clinical trials of selegiline there is no evidence for enhanced cardiovascular risk (Parkinson Study Group, 1989, 1993), as is true also when selegiline is compared to treatment based on LD and DA receptor agonists; however these drugs have never been studied in head-to-head comparison. Long term trials have shown that 30–40% of the daily LD dose can be reduced when the drug is combined with selegiline (Birkmayer et al., 1975; Myllyla et al., 1997). One interesting hypothesis about the good beneficial effect of MAO-B inhibitors is that PEA, which increases in brain after selegiline treatment (Reynolds et al., 1978), may have DA release-promoting activity and by this way contribute to the positive effects on motor features and behavior.

In 1989 Tetrud and Langston published a clinical study based on the discovery that selegiline blocked the development of MPTP-induced parkinsonism in laboratory animals, in which they showed that in 22 PD patients medicated with selegiline and 22 who received placebo the necessity to add LD (rescue drug) occurred in the placebo group after 312.1 days and in the selegiline group after 548.9 days (Tetrud and Langston, 1989). In the DATATOP (Deprenyl and Tocopherol Antioxidative Treatment of Parkinsonism) publications (Parkinson Study Group, 1989, 1993) after 12 months of monotherapy with selegiline or placebo (800 patients), 47% in the placebo group had commenced LD therapy while in the selegiline group only 26%. In addition it is important to mention that in the placebo group the median length of time before patients needed LD was 454 days and in the selegiline group 719 days. In selegiline-treated patients the need for addition of LD therapy was postponed by ~9 months.

Shoulson et al. (2002) published interesting data concerning long term follow up (7 years) of patients in the DATATOP group treated with LD and selegiline, as well as those who received placebo after 3–5 years treatment with selegiline. The conclusions revealed that the wearing-off phenomenon did not improve in the selegiline-treated patients but these patients had less on-off phenomenon or freezing of gait and better motor features with lower total UPDRS (Unified Parkinson's Disease Rating Scores) score. Activities of daily living (ADL), motor scores, LD dosages and use of DA agonists were significantly reduced in the selegiline groups. On the other hand, patients in the placebo group had less dyskinesias than those on selegiline.

It is important to note that, in DATATOP and its extension study it was impossible to distinguish between potential disease–modifying (i.e., neuroprotective) and symptomatic benefits of treatment (Parkinson Study Group, 1996a,b, 1989, 1993; Shoulson et al., 2002). In another study (SELEDO), Przuntek et al. (1999) showed that the length of time in which PD patients treated with selegiline required an increase of 50% in LD dose was 4.9 years, while in placebo-treated patients it was 2.6 years. Myllyla et al. (1992, 1997) reported similar results.

The DATATOP results along with other earlier studies demonstrated a modest symptomatic benefit with selegiline with no significant difference between selegiline and placebo in the occurrence of cardiovascular and other serious adverse events. Long-term post-marketing data, however, have revealed that orthostatic hypotension and hallucinations are seen frequently in selegiline-treated patients, mainly in combination with LD (Perez-Lloret et al., 2013).

A trial in 782 PD patients by the Parkinson's Disease Research Group of the United Kingdom (Lees, 1995; Ben-Shlomo et al., 1998) concluded that the addition of selegiline to ongoing LD therapy provided no additional clinical benefit but was associated with increased motor complications and increased mortality. A follow-up study from the same group examined postural hypotension in a sub-group of patients and found that in 8 out of 22 patients postural hypotension was exacerbated when on selegiline, and this effect was abolished following withdrawal of the drug (Churchyard et al., 1999). When experts who did not participate in the study analyzed the results (Olanow et al., 1998) they found that the conclusions reported were not correct and in fact there was no increase in mortality with selegiline treatment whether administered alone or in combination with LD (Olanow et al., 1998). Considering selegiline treatment in randomized studies it is important to mention a 157-patient, randomized controlled Swedish study of selegiline long-term effects when used in early PD either as monotherapy or in combination with LD. This trial showed that at 7 years, selegiline-treated patients had slower disease progression than their counterparts as measured with UPDRS score. This outcome was observed in the selegiline monotherapy PD patients as well as in those treated in addition with LD (Palhagen et al., 2006), however also in this study, the symptomatic effect of selegiline precludes drawing conclusions about disease modification.

In general, summarizing most of the reports selegiline is well tolerated. Side effects/adverse effects like nausea, vomiting, sleeplessness, dry mouth, orthostatic hypotension and dyskinesias have all been observed in the range of 2–5% of PD patients (Parkinson Study Group, 1993; Reichmann et al., 2000) which is comparable to placebo. Other side effects like headaches, palpitations, dyspneas, confusion, edema, micturition dysfunction, loss of appetite and anxiety have an incidence below 2% (Reichmann et al., 2000).

Waters et al (Waters et al., 2004) examined the effect of Zydis selegiline in a 3 month, randomized, placebo-controlled study in PD patients experiencing motor fluctuations in response to LD and found that it reduced off time by 2.2 h (compared with 0.6 h in the placebo group), without any increase in drug-related adverse events.

### Rasagiline

Rasagiline is a potent, selective, irreversible inhibitor of MAO-B and in contrast to selegiline has no amphetamine-like metabolites (Finberg et al., 1999). Given in disease models relevant to PD (Bar-Am et al., 2010), rasagiline showed good antiparkinsonian and motor restoration activity as well as neuroprotection properties, and its major metabolite 1-aminoindan is also neuroprotective (see above section on Neuroprotection).

In a 10 week, randomized placebo-controlled pilot (phase 2) trial of rasagiline in patients with early, untreated PD, a dose of up to 4 mg/day was well tolerated. There were no cases of hypertension, bradycardia or other cardiovascular adverse experiences (Marek et al., 1997). One of the early randomized clinical studies comparing rasagiline vs. placebo for advanced PD patients was published in 2000 (Rabey et al., 2000). In this study researchers planned to evaluate the safety, tolerability and clinical effect of rasagiline as adjunct therapy with LD, in a multicenter, double blind, placebo-controlled parallel group study (0.5, 1, and 2 mg/day) lasting 12 weeks, in 70 patients with PD (mean age 57.4 years, mean disease duration 5.7 years; 32 patients had motor fluctuations). A beneficial clinical effect was observed in fluctuating patients treated with rasagiline (all doses) and was expressed as a decrease in total UPDRS score (by 23% in rasagiline-, 8.5% in placebo-treated subjects). The anti-Parkinsonian effect of rasagiline was still evident 6 weeks after stopping treatment at all dose levels. The incidence of adverse effects with rasagiline was similar to those on placebo. Determination of platelet MAO activity (MAO-B) showed nearly complete MAO-B inhibition at all rasagiline dose levels. This study showed that rasagiline (up to 2 mg/day) is well-tolerated and has a beneficial clinical effect in fluctuating patients with PD when given together with chronic LD therapy.

In the TEMPO (Rasagiline mesylate in Early Monotherapy for Parkinson's disease Outpatients) study (Parkinson Study Group, 2002), 404 *de novo* (untreated) PD patients received either placebo (*n* = 138), rasagiline 1 mg per day (*n* = 134) or rasagiline 2 mg per day (*n* = 132) for 26 weeks. All the patients receiving rasagiline showed statistically significant improvement compared with placebo in the mean change from baseline of the Unified Parkinsons's Disease Rating Scale (UPDRS part I-III) by 4.2 and 3.6 points, in 1 and 2 mg/day groups respectively. Patients on rasagiline showed also a significant and beneficial effect for quality of life as assessed by the Parkinson's Disease Quality of Life (PD-QUALIF) scale.

Considering the results of preclinical studies which suggested that rasagiline may modify the progression of PD (Maruyama et al., 2001; Akao et al., 2002), the TEMPO study was extended into a delayed start trial comparing the effects of early and later initiation of rasagiline on disease progression (Parkinson Study Group, 2004). Three hundred seventy–one subjects from the TEMPO study were included in the 1-year efficacy analysis. Patients who had received rasagiline 1 or 2 mg/day for 6 months received the same dose for a further 6 months, while those who had been treated with placebo for 6 months were given rasagiline 2 mg/day for a further 6 months. This design was adopted in order to compensate for the symptomatic effect of rasagiline, which prevented concluding that the drug had a disease-modifying action when rasagiline-treated patients were compared with those treated with placebo. In this delayed-start trial, it was possible to compare patients who had received rasagiline 2 mg/day for 12 months with those who had received this dose for only 6 months, but all were receiving the drug at the time of neurological assessment at 12 months from start. The result of this trial was that patients who had received rasagiline 2 mg/day for 12 months had a 2.29-unit smaller increase in UPDRS score than those who were treated with rasagiline 2 mg/day for 6 months (*P* = 0.01). This delayed-start analysis suggested that rasagiline could have a disease-modifying activity.

The ability of rasagiline to improve LD response in more advanced PD patients with motor fluctuations was studied in LARGO (Lasting effect in Adjunct therapy with Rasagiline Given Once daily) (Rascol et al., 2005). In this study 231 individuals, received rasagiline (1 mg daily), 229 received placebo, and 227 received entacapone (200 mg daily). All were treated with LD and a decarboxylase inhibitor. The primary outcome was change in total daily off-time. Other measures included the (CGI) score and the UPDRS scores. Both rasagiline and entacapone reduced mean daily off-time (−1.18 h rasagiline and −1.2 h entacapone vs. placebo −0.4). A significant improvement in clinical global improvement (CGI) scores, and activities of daily living during off-time and motor function during on time was seen with both rasagiline and entacapone. Adverse effects were similar in all groups.

To date the majority of PD clinical studies conducted in patients with motor fluctuations have reported the duration of OFF time during the day with limited evaluation of the severity of PD symptoms during ON time. The irreversible nature of the binding of rasagiline to the essential cofactor of the active site of the MAO-B enzyme means that the duration of its therapeutic action is independent of the drug's half-life and is instead determined by the regeneration rate of MAO-B (Thebault et al., 2004). The Largo study included a subgroup of patients, “UPDRS motor OFF substudy,” for which there was a separate informed consent form. Patients included in this sub-study, were receiving optimum LD + decarboxylase inhibitor therapy, were stable for at least 14 days before study start, and experienced motor fluctuations in which they were in OFF state for at least 1 h every day not including morning akinesia. Additional antiparkinsonian therapy was accepted, with the exception of selegiline, tolcapone and previous treatment with entacapone. At the start of the 18 weeks study patients were randomly assigned to receive placebo, rasagiline 1 mg or entacapone 200 mg in addition to LD and decarboxylase inhibitor. The LD dose could be reduced during the first 6 weeks if dyskinesia worsened. Thereafter the LD dose remained the same for the final 12 weeks of the study (Stocchi and Rabey, 2011). Treatment with rasagiline produced a significant improvement over placebo of 5.64 units in UPDRS motor OFF score (*P* = 0.013 vs. placebo). By contrast the effect of adjunct entacapone was not significant (*P* = 0.14 vs. placebo). Retrospective analysis using the Bonferroni correction of UPDRS motor subdomains further revealed that rasagiline but not entacapone, significantly improved bradykinesia (*p* < 0.001) and showed a trend for improvement in facial expression, speech and axial impairment during OFF time.

In Olanow et al. (2009) conducted a double blind study, with early and delayed start rasagiline (Attenuation of Disease Progression with Rasagiline Given Once-daily, ADAGIO), in order to establish more substantially whether this drug has disease-modifying effect in PD. A total of 1091 untreated PD patients participated in the start of the study; 273 early start rasagiline 1 mg/day, 270 delayed start rasagiline 1 mg/day, 273 early start rasagiline 2 mg/day, and 275 delayed start rasagiline 2 mg/daily. The early start group received the drug for 72 weeks, the delayed start group received placebo for 36 weeks followed by rasagiline for a further 36 weeks.

**Figure 1:**
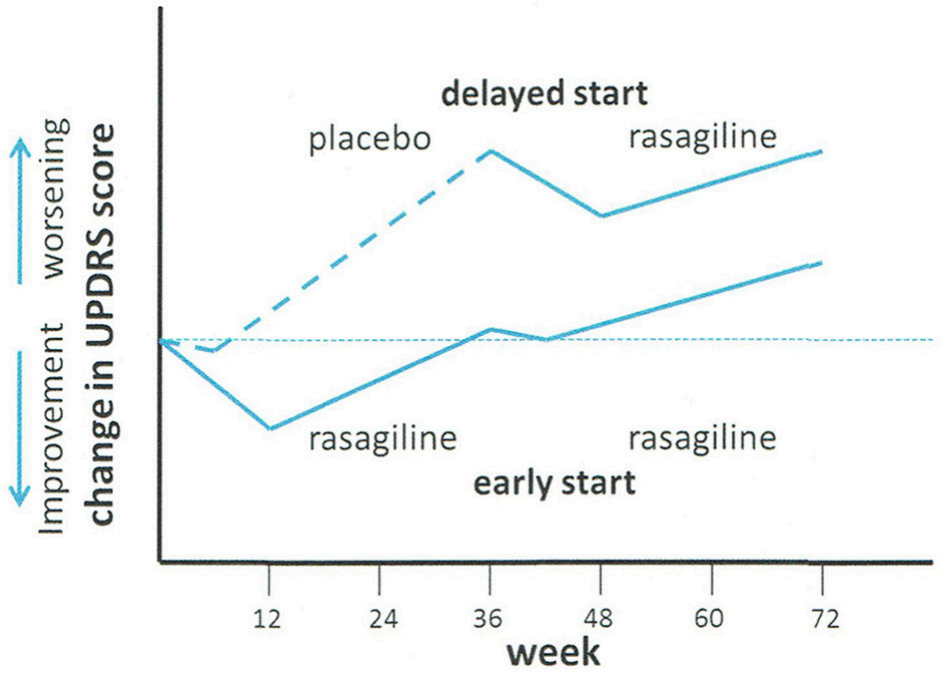
**Schematic of ADAGIO delayed start neuroprotection trial of rasagiline**. Treatment with placebo (delayed start group) or rasagiline (early start group) was commenced at time 0. At week 36, the placebo group was changed to rasagiline, and the rasagiline group continued with rasagiline. Adapted from Olanow et al. (2009).

The putative result required for disease-modification is shown diagrammatically in Figure 1

Three endpoints were required to be met in order to permit a positive result: (a) increased slope of the decline in UPDRS in the placebo group with respect to the rasagiline group in the first phase of the study (i.e., week 12–36); (b) a difference between the early- and delayed-start groups in the change in UPDRS score between weeks 12 and 72, and (c) similarity of the slopes of change in UPDRS score with time in the period between weeks 48 and 72. Finite values of UPDRS were fixed for the three endpoints. The conclusions of the study were: “Early treatment with rasagiline at a dose of 1 mg per day provided benefits that were consistent with a possible disease-modifying effect, but early treatment with rasagiline at a dose of 2 mg per day did not. Because the two doses were associated with different outcomes, the study results must be interpreted with caution” (Churchyard et al., 1999).

Following publication of the Adagio results, there has been much discussion about the significance of the data. Rascol et al. published in 2011 a secondary and *post-hoc* comment on the ADAGIO study (Rascol et al., 2011). In addition to the criterion of UPDRS score, they included changes in non-motor experiences of daily living (ADL), fatigue scales, the need for additional antiparkinsonian therapy, and (UPDRS) subscores, in the data assessment. In addition to the finding that rasagiline therapy delayed the need for additional antiparkinsonian drugs, and improved ADL scores in the 1 mg/daily early start group, they showed that the rate of deterioration in UPDRS scores correlated with baseline scores, patients with low baseline scores deteriorating slower than those with higher baseline scores. A difference in the distribution of low and high baseline UPDRS scores between the 1 and 2 mg daily groups could have contributed to the lack of difference between early and late start groups at the 2 mg dose level.

In 2014, Jankovic et al. (2014) published another *post-hoc* analysis from the ADAGIO study in which they examined the responses of patients to rasagiline 1 mg/day (*n* = 288) with those to placebo (*n* = 588) on key motor symptoms at 36 weeks. In the rasagiline group, significantly better tremor, bradykinesia, rigidity and postural-instability-gait difficulty scores were seen at week 36 by comparison with placebo. While the placebo group deteriorated from baseline by 2.6 points UPDRS at week 36, patients in the rasagiline group improved initially but then returned to baseline values at week 36. At week 72 patients who had received continuous monotherapy with rasagiline experienced a worsening of only 1.6 points. The conclusions of this analysis were that treatment with rasagiline maintained motor function at baseline values for at least a year with significant benefit observed in all key PD motor symptoms.

### Safinamide

Safinamide, an orally active alpha-aminoamide derivative, is a novel reversible and highly selective MAO-B inhibitor which is efficacious as add-on therapy to DA agonists in early-stage PD (Stocchi et al., 2004, 2012) and as adjunct to LD in mid- to late-stage PD (Borgohain et al., 2014). In addition to MAO inhibition, the molecule possesses additional pharmacological properties, including state-dependent blockade of voltage-gated sodium and calcium channels, and inhibition of glutamate release in rat hippocampal synaptosomes (Caccia et al., 2006; Stocchi et al., 2006). These properties may be responsible for its demonstrated neuroprotective effect in laboratory animals.

The use of safinamide as adjunct therapy to LD was investigated in mid- to late-stage PD patients with motor fluctuations (Waters et al., 2004). In an initial 6 month trial, safinamide 50 mg (*n* = 197) or 100 mg (*n* = 195) daily was studied in relation to placebo (*n* = 197) and was found to significantly increase ON time without increasing dyskinesia. The study was then continued in the same patient population for an additional 18 months. In the final evaluation of the 2 year period, both safinamide groups had a significant increase in ON-time compared to placebo, which was maintained over the period between 24 and 102 weeks. A non-significant reduction in Dyskinesia Rating Score (DRS) was found for both safinamide groups, but in considering this result, 74% of the population had no to mild dyskinesia at baseline. In the case of the sub-group with more severe dyskinesia (DRS > 4, 36% of the population), safinamide caused a reduction in DRS in relation to placebo, which was significant (*P* = 0.0317) at the 100 mg daily dose level. Other benefits noted by the investigators in the safinamide group included improvements in ADL, depression, clinical status and quality of life (Borgohain et al., 2014). In an efficacy study reported by Schapira et al. (2013) patients received 100 mg, 200 mg safinamide or placebo added to LD, or DA agonists. In their study the conclusions were that the safinamide group did not attain the primary endpoint of increase in time required for additional drug therapy, however *post hoc* analysis showed that safinamide was effective in PD therapy in combination with DA agonists.

A number of research groups are aiming to develop other MAO-A and MAO-B reversible inhibitors. A series of chalcone derivatives with reversible MAO-A and MAO-B selective properties was recently described (Minders et al., 2015). The IC_50_ of the most potent MAO-B inhibitor in this series was 0.067 μM, by comparison with 0.098 μM for safinamide. Further data on *in vivo* metabolism and efficacy will be required to see whether this compound has therapeutic potential.

## Conclusions

Today there is a broad spectrum of therapeutic possibilities for the utilization of MAO-A and -B inhibitors, for the management of PD, and also for the treatment of depression. Novel routes of administration, as well as pro-drugs which are converted to active inhibitors by brain enzymes, are promising directions for development of MAOI with selective action in the brain in order to avoid cheese effect. New drug developments which combine different types of activity in the same molecule, and can possibly be effective in more than one disease condition, may be useful in treatment of both neuropsychiatric and neurological disorders, and mechanism-based drug combinations may improve efficacy in PD and other diseases

## Author contributions

JF: Initiated this review, contributed introductory section, mechanisms and section on psychiatric uses, reviewed and submitted article; JR: Contributed section on Parkinson's Disease.

### Conflict of interest statement

JF was a co-developer of rasagiline and receives financial gain from sales of this drug. Such financial gain did not in any way influence the thoughts and opinions expressed in this article. The other author declares that the research was conducted in the absence of any commercial or financial relationships that could be construed as a potential conflict of interest.

## References

[B1] Akao Y. Maruyama W. Yi H. Shamoto-Nagai M. Youdim M. B. Naoi M. (2002). An anti-Parkinson's disease drug, N-propargyl-1(R)-aminoindan (rasagiline), enhances expression of anti-apoptotic bcl-2 in human dopaminergic SH-SY5Y cells. Neurosci. Lett. 326, 105–108. 10.1016/S0304-3940(02)00332-4 12057839

[B2] Akhondzadeh S. Tavakolian R. Davari-Ashtiani R. Arabgol F. Amini H. (2003). Selegiline in the treatment of attention deficit hyperactivity disorder in children: a double blind and randomized trial. Prog. Neuropsychopharmacol. Biol. Psychiatry 27, 841–845. 10.1016/S0278-5846(03)00117-9 12921918

[B3] Alia-Klein N. Goldstein R. Z. Kriplani A. Logan J. Tomasi D. Williams B.. (2008). Brain monoamine oxidase A activity predicts trait aggression. J. Neurosci. 28, 5099–5104. 10.1523/JNEUROSCI.0925-08.2008 18463263 PMC2430409

[B4] Aluf Y. Vaya J. Khatib S. Finberg J. P. (2011). Alterations in striatal oxidative stress level produced by pharmacological manipulation of dopamine as shown by a novel synthetic marker molecule. Neuropharmacology 61, 87–94. 10.1016/j.neuropharm.2011.03.006 21414328

[B5] Aluf Y. Vaya J. Khatib S. Loboda Y. Finberg J. P. (2013). Selective inhibition of monoamine oxidase A or B reduces striatal oxidative stress in rats with partial depletion of the nigro-striatal dopaminergic pathway. Neuropharmacology 65, 48–57. 10.1016/j.neuropharm.2012.08.023 22982254

[B6] Arnett C. D. Fowler J. S. MacGregor R. R. Schlyer D. J. Wolf A. P. Langstrom B.. (1987). Turnover of brain monoamine oxidase measured *in vivo* by positron emission tomography using L-[11C]deprenyl. J. Neurochem. 49, 522–527. 10.1111/j.1471-4159.1987.tb02895.x 3110375

[B7] Azzaro A. J. Vandenberg C. M. Blob L. F. Kemper E. M. Sharoky M. Oren D. A.. (2006). Tyramine pressor sensitivity during treatment with the selegiline transdermal system 6 mg/24 h in healthy subjects. J. Clin. Pharmacol. 46, 933–944. 10.1177/0091270006289852 16855078

[B8] Azzaro A. J. Ziemniak J. Kemper E. Campbell B. J. VanDenBerg C. (2007). Pharmacokinetics and absolute bioavailability of selegiline following treatment of healthy subjects with the selegiline transdermal system (6 mg/24 h): a comparison with oral selegiline capsules. J. Clin. Pharmacol. 47, 1256–1267. 10.1177/0091270007304779 17715422

[B9] Badinter F. Amit T. Bar-Am O. Youdim M. B. Weinreb O. (2015). Beneficial behavioral, neurochemical and molecular effects of 1-(R)-aminoindan in aged mice. Neuropharmacology 99, 264–272. 10.1016/j.neuropharm.2015.05.041 26087462

[B10] Baker G. B. Wong J. T. Yeung J. M. Coutts R. T. (1991). Effects of the antidepressant phenelzine on brain levels of gamma-aminobutyric acid (GABA). J. Affect. Disord. 21, 207–211. 10.1016/0165-0327(91)90041-P 1648582

[B11] Bar-Am O. Amit T. Kupershmidt L. Aluf Y. Mechlovich D. Kabha H.. (2015). Neuroprotective and neurorestorative activities of a novel iron chelator-brain selective monoamine oxidase-A/monoamine oxidase-B inhibitor in animal models of Parkinson's disease and aging. Neurobiol. Aging 36, 1529–1542. 10.1016/j.neurobiolaging.2014.10.026 25499799

[B12] Bar-Am O. Weinreb O. Amit T. Youdim M. B. (2010). The neuroprotective mechanism of 1-(R)-aminoindan, the major metabolite of the anti-parkinsonian drug rasagiline. J. Neurochem. 112, 1131–1137. 10.1111/j.1471-4159.2009.06542.x 20002521

[B13] Ben-Shlomo Y. Churchyard A. Head J. Hurwitz B. Overstall P. Ockelford J.. (1998). Investigation by Parkinson's disease research group of United Kingdom into excess mortality seen with combined levodopa and selegiline treatment in patients with early, mild Parkinson's disease: further results of randomised trial and confidential inquiry. BMJ 316:1191–1196. 10.1136/bmj.316.7139.1191 9583926 PMC28519

[B14] Bieck P. R. Antonin K. H. (1988). Oral tyramine pressor test and the safety of monoamine oxidase inhibitor drugs: comparison of brofaromine and tranylcypromine in healthy subjects. J. Clin. Psychopharmacol. 8, 237–245. 10.1097/00004714-198808000-00002 3209716

[B15] Bieck P. R. Antonin K. H. (1994). Tyramine potentiation during treatment with MAOIs, in Clinical Advances in Monoamine Oxidase Inhibitor Therapies, ed Kennedy S. H. (Washington, DC: American Psychiatric Press), 83–110.

[B16] Binda C. Hubalek F. Li M. Herzig Y. Sterling J. Edmondson D. E.. (2005). Binding of rasagiline-related inhibitors to human monoamine oxidases: a kinetic and crystallographic analysis. J. Med. Chem. 48, 8148–8154. 10.1021/jm0506266 16366596 PMC2519603

[B17] Binda C. Newton-Vinson P. Hubalek F. Edmondson D. E. Mattevi A. (2002). Structure of human monoamine oxidase B, a drug target for the treatment of neurological disorders. Nat. Struct. Biol. 9, 22–26. 10.1038/nsb732 11753429

[B18] Binda C. Wang J. Pisani L. Caccia C. Carotti A. Salvati P.. (2007). Structures of human monoamine oxidase B complexes with selective noncovalent inhibitors: safinamide and coumarin analogs. J. Med. Chem. 50, 5848–5852. 10.1021/jm070677y 17915852

[B19] Birkmayer W. Riederer P. Youdim M. B. Linauer W. (1975). The potentiation of the anti akinetic effect after L-dopa treatment by an inhibitor of MAO-B, Deprenil. J. Neural. Transm. 36, 303–326. 10.1007/BF01253131 1172524

[B20] Blob L. F. Sharoky M. Campbell B. J. Kemper E. M. Gilmor M. G. VanDenberg C. M.. (2007). Effects of a tyramine-enriched meal on blood pressure response in healthy male volunteers treated with selegiline transdermal system 6 mg/24 hour. CNS Spectr. 12, 25–34. 10.1017/S1092852900020496 17192761

[B21] Borgohain R. Szasz J. Stanzione P. Meshram C. Bhatt M. H. Chirilineau D.. (2014). Two-year, randomized, controlled study of safinamide as add-on to levodopa in mid to late Parkinson's disease. Mov. Disord. 29, 1273–1280. 10.1002/mds.25961 25044402

[B22] Buccafusco J. J. Terry A. V. Jr. Goren T. Blaugrun E. (2003). Potential cognitive actions of (n-propargly-(3r)-aminoindan-5-yl)-ethyl, methyl carbamate (tv3326), a novel neuroprotective agent, as assessed in old rhesus monkeys in their performance of versions of a delayed matching task. Neuroscience 119, 669–678. 10.1016/S0306-4522(02)00937-5 12809688

[B23] Caccia C. Maj R. Calabresi M. Maestroni S. Faravelli L. Curatolo L.. (2006). Safinamide: from molecular targets to a new anti-Parkinson drug. Neurology 67, S18–23. 10.1212/WNL.67.7_suppl_2.S18 17030736

[B24] Carlile G. W. Chalmers-Redman R. M. Tatton N. A. Pong A. Borden K. E. Tatton W. G. (2000). Reduced apoptosis after nerve growth factor and serum withdrawal: conversion of tetrameric glyceraldehyde-3-phosphate dehydrogenase to a dimer. Mol. Pharmacol. 57:2–12. 10617673

[B25] Carlo P. Del Rio M. Violani E. Sciaba L. Picotti G. B. (1996). Influence of culture conditions on monoamine oxidase A and B activity in rat astrocytes. Cell Biochem. Funct. 14, 19–25. 10.1002/cbf.645 8907250

[B26] Churchyard A. Mathias C. J. Boonkongchuen P. Lees A. J. (1997). Autonomic effects of selegiline: possible cardiovascular toxicity in Parkinson's disease. J. Neurol. Neurosurg. Psychiatry. 63, 228–234. 10.1136/jnnp.63.2.228 9285463 PMC2169684

[B27] Churchyard A. Mathias C. J. Lees A. J. (1999). Selegiline-induced postural hypotension in Parkinson's disease: a longitudinal study on the effects of drug withdrawal. Mov. Disord. 14, 246–251. 10.1002/1531-8257(199903)14:2<246::AID-MDS1008>3.0.CO;2-P 10091617

[B28] Clarke A. Brewer F. Johnson E. S. Mallard N. Hartig F. Taylor S.. (2003b). A new formulation of selegiline: improved bioavailability and selectivity for MAO-B inhibition. J. Neural. Transm. (Vienna). 110, 1241–1255. 10.1007/s00702-003-0036-4 14628189

[B29] Clarke A. Johnson E. S. Mallard N. Corn T. H. Johnston A. Boyce M.. (2003a). A new low-dose formulation of selegiline: clinical efficacy, patient preference and selectivity for MAO-B inhibition. J. Neural. Transm. (Vienna). 110, 1257–1271. 10.1007/s00702-003-0042-6 14628190

[B30] Cohen L. J. Sclar D. A. (2012). Issues in adherence to treatment with monoamine oxidase inhibitors and the rate of treatment failure. J. Clin. Psychiatry 73(Suppl. 1), 31–36. 10.4088/JCP.11096su1c.05 22951240

[B31] Coyle J. T. Snyder S. H. (1969). Antiparkinsonian drugs: inhibition of dopamine uptake in the corpus striatum as a possible mechanism of action. Science 166, 899–901. 10.1126/science.166.3907.899 5345207

[B32] De Colibus L. Li M. Binda C. Lustig A. Edmondson D. E. Mattevi A. (2005). Three-dimensional structure of human monoamine oxidase A (MAO A): relation to the structures of rat MAO A and human MAO B. Proc. Natl. Acad. Sci. U.S.A. 102, 12684–12689. 10.1073/pnas.0505975102 16129825 PMC1200291

[B33] Denney R. M. Denney C. B. (1985). An update on the identity crisis of monoamine oxidase: new and old evidence for the independence of MAO A and B. Pharmacol. Ther. 30, 227–258. 10.1016/0163-7258(85)90050-6 3916286

[B34] Elkashef A. Fudala P. J. Gorgon L. Li S. H. Kahn R. Chiang N.. (2006). Double-blind, placebo-controlled trial of selegiline transdermal system (STS) for the treatment of cocaine dependence. Drug. Alcohol. Depend. 85, 191–197. 10.1016/j.drugalcdep.2006.04.010 16730924

[B35] Elsworth J. D. Sandler M. Lees A. J. Ward C. Stern G. M. (1982). The contribution of amphetamine metabolites of (−)-deprenyl to its antiparkinsonian properties. J. Neural. Transm. 54, 105–110. 10.1007/BF01249283 6809891

[B36] Fearnley J. M. Lees A. J. (1991). Ageing and Parkinson's disease: substantia nigra regional selectivity. Brain 114(Pt 5), 2283–2301. 10.1093/brain/114.5.2283 1933245

[B37] Finberg J. P. (2014). Update on the pharmacology of selective inhibitors of MAO-A and MAO-B: focus on modulation of CNS monoamine neurotransmitter release. Pharmacol. Ther. 143, 133–152. 10.1016/j.pharmthera.2014.02.010 24607445

[B38] Finberg J. P. Gillman P. K. (2011). Selective inhibitors of monoamine oxidase type b and the cheese effect, in International Review of Neurobiology, eds Youdim M. B. Riederer P. (Burlington, VT: Academic Press), 169–190. 10.1016/B978-0-12-386467-3.00009-1 21971008

[B39] Finberg J. P. Lamensdorf I. Weinstock M. Schwartz M. Youdim M. B. (1999). Pharmacology of rasagiline (N-propargyl-1R-aminoindan). Adv. Neurol. 80, 495–499. 10410762

[B40] Finberg J. P. Takeshima T. Johnston J. M. Commissiong J. W. (1998). Increased survival of dopaminergic neurons by rasagiline, a monoamine oxidase B inhibitor. Neuroreport 9, 703–707. 10.1097/00001756-199803090-00026 9559942

[B41] Finberg J. P. Tenne M. (1982). Relationship between tyramine potentiation and selective inhibition of monoamine oxidase types A and B in the rat vas deferens. Br. J. Pharmacol. 77, 13–21. 10.1111/j.1476-5381.1982.tb09263.x 6127132 PMC2044656

[B42] Finberg J. P. Youdim M. B. (2002). Pharmacological properties of the anti-Parkinson drug rasagiline; modification of endogenous brain amines, reserpine reversal, serotonergic and dopaminergic behaviours. Neuropharmacology 43, 1110–1118. 10.1016/S0028-3908(02)00216-2 12504917

[B43] Freedman N. M. Mishani E. Krausz Y. Weininger J. Lester H. Blaugrund E.. (2005). *In vivo* measurement of brain monoamine oxidase B occupancy by rasagiline, using (11)C-l-deprenyl and PET. J. Nucl. Med. 46, 1618–1624. 16204711

[B44] Gahr M. Schonfeldt-Lecuona C. Kolle M. A. Freudenmann R. W. (2013). Withdrawal and discontinuation phenomena associated with tranylcypromine: a systematic review. Pharmacopsychiatry 46:123–129. 10.1055/s-0032-1333265 23359339

[B45] Gal S. Zheng H. Fridkin M. Youdim M. B. (2005). Novel multifunctional neuroprotective iron chelator-monoamine oxidase inhibitor drugs for neurodegenerative diseases. In vivo selective brain monoamine oxidase inhibition and prevention of MPTP-induced striatal dopamine depletion. J. Neurochem. 95, 79–88. 10.1111/j.1471-4159.2005.03341.x 16181414

[B46] Gillman P. K. (2006). A review of serotonin toxicity data: implications for the mechanisms of antidepressant drug action. Biol. Psychiatry 59, 1046–1051. 10.1016/j.biopsych.2005.11.016 16460699

[B47] Gillman P. K. (2011). Advances pertaining to the pharmacology and interactions of irreversible nonselective monoamine oxidase inhibitors. J. Clin. Psychopharmacol. 31, 66–74. 10.1097/JCP.0b013e31820469ea 21192146

[B48] Goelman G. Ilinca R. Zohar I. Weinstock M. (2014). Functional connectivity in prenatally stressed rats with and without maternal treatment with ladostigil, a brain-selective monoamine oxidase inhibitor. Eur. J. Neurosci. 40, 2734–2743. 10.1111/ejn.12621 24862938

[B49] Goldberg J. F. Thase M. E. (2013). Monoamine oxidase inhibitors revisited: what you should know. J. Clin. Psychiatry 74, 189–191. 10.4088/JCP.12ac08299 23473352

[B50] Goren T. Adar L. Sasson N. Weiss Y. M. (2010). Clinical pharmacology tyramine challenge study to determine the selectivity of the monoamine oxidase type B (MAO-B) inhibitor rasagiline. J. Clin. Pharmacol. 50, 1420–1428. 10.1177/0091270010369674 20445015

[B51] Harris D. S. Everhart T. Jacob P. IIIrd Lin E. Mendelson J. E. Jones R. T. (2009). A phase 1 trial of pharmacologic interactions between transdermal selegiline and a 4-hour cocaine infusion. BMC Clin. Pharmacol. 9:13. 10.1186/1472-6904-9-13 19646280 PMC2731040

[B52] Harvey B. H. Duvenhage I. Viljoen F. Scheepers N. Malan S. F. Wegener G.. (2010). Role of monoamine oxidase, nitric oxide synthase and regional brain monoamines in the antidepressant-like effects of methylene blue and selected structural analogues. Biochem. Pharmacol. 80, 1580–1591. 10.1016/j.bcp.2010.07.037 20699087

[B53] Heijnen W. T. De Fruyt J. Wierdsma A. I. Sienaert P. Birkenhager T. K. (2015). Efficacy of tranylcypromine in bipolar depression: a systematic review. J. Clin. Psychopharmacol. 35, 700–705. 10.1097/JCP.0000000000000409 26479223

[B54] Hill M. N. Ho W. S. Hillard C. J. Gorzalka B. B. (2008). Differential effects of the antidepressants tranylcypromine and fluoxetine on limbic cannabinoid receptor binding and endocannabinoid contents. J. Neural. Transm. 115, 1673–1679. 10.1007/s00702-008-0131-7 18974922 PMC2992975

[B55] Ho M. C. Cherng C. G. Tsai Y. P. Chiang C. Y. Chuang J. Y. Kao S. F.. (2009). Chronic treatment with monoamine oxidase-B inhibitors decreases cocaine reward in mice. Psychopharmacology (Berl). 205, 141–149. 10.1007/s00213-009-1524-5 19343328

[B56] Hung C. F. Lung F. W. Hung T. H. Chong M. Y. Wu C. K. Wen J. K.. (2012). Monoamine oxidase A gene polymorphism and suicide, an association study and meta-analysis. J. Affect. Disord. 136, 643–649. 10.1016/j.jad.2011.10.013 22041522

[B57] Jankovic J. Berkovich E. Eyal E. Tolosa E. (2014). Symptomatic efficacy of rasagiline monotherapy in early Parkinson's disease: *post-hoc* analyses from the ADAGIO trial. Parkinsonism Relat. Disord. 20, 640–643. 10.1016/j.parkreldis.2014.02.024 24637126

[B58] Jenner P. (2003). Oxidative stress in Parkinson's disease. Ann. Neurol. 53(Suppl. 3), S26–36 discussion S36–28. 10.1002/ana.10483 12666096

[B59] Jenner P. Langston J. W. (2011). Explaining ADAGIO: a critical review of the biological basis for the clinical effects of rasagiline. Mov. Disord. 26, 2316–2323. 10.1002/mds.23926 21953831

[B60] Kolla N. J. Matthews B. Wilson A. A. Houle S. Bagby R. M. Links P.. (2015). Lower monoamine oxidase-a total distribution volume in impulsive and violent male offenders with antisocial personality disorder and high psychopathic traits: an [(11)C] harmine positron emission tomography study. Neuropsychopharmacology 40, 2596–2603. 10.1038/npp.2015.106 26081301 PMC4569949

[B61] Korchounov A. Winter Y. Rossy W. (2012). Combined beneficial effect of rasagiline on motor function and depression in *de novo* PD. Clin. Neuropharmacol. 35, 121–124. 10.1097/WNF.0b013e31823b1da8 22561875

[B62] Lader M. H. Sakalis G. Tansella M. (1970). Interactions between sympathomimetic amines and a new monoamine oxidase inhibitor. Psychopharmacologia 18:118–123. 10.1007/BF00402391 4943186

[B63] Laine K. Anttila M. Huupponen R. Maki-Ikola O. Heinonen E. (2000). Multiple-dose pharmacokinetics of selegiline and desmethylselegiline suggest saturable tissue binding. Clin. Neuropharmacol. 23, 22–27. 10.1097/00002826-200001000-00005 10682227

[B64] Lamensdorf I. Porat S. Simantov R. Finberg J. P. (1999). Effect of low-dose treatment with selegiline on dopamine transporter (DAT) expression and amphetamine-induced dopamine release *in vivo*. Br. J. Pharmacol. 126, 997–1002. 10.1038/sj.bjp.0702389 10193780 PMC1571229

[B65] Lamensdorf I. Youdim M. B. Finberg J. P. (1996). Effect of long-term treatment with selective monoamine oxidase A and B inhibitors on dopamine release from rat striatum *in vivo*. J. Neurochem. 67, 1532–1539. 10.1046/j.1471-4159.1996.67041532.x 8858937

[B66] Lang A. E. Lozano A. M. (1998a). Parkinson's disease. First of two parts. N. Engl. J. Med. 339, 1044–1053. 10.1056/NEJM199810083391506 9761807

[B67] Lang A. E. Lozano A. M. (1998b). Parkinson's disease. Second of two parts. N. Engl. J. Med. 339, 1130–1143. 10.1056/NEJM199810153391607 9770561

[B68] Lee M. G. Wynder C. Schmidt D. M. McCafferty D. G. Shiekhattar R. (2006). Histone H3 lysine 4 demethylation is a target of nonselective antidepressive medicataions. Chem. Biol. 13, 563–567. 10.1016/j.chembiol.2006.05.004 16793513

[B69] Lees A. J. (1995). Comparison of therapeutic effects and mortality data of levodopa and levodopa combined with selegiline in patients with early, mild Parkinson's disease. Parkinson's Disease research group of the United Kingdom. BMJ 311:1602–1607. 10.1136/bmj.311.7020.1602 8555803 PMC2551499

[B70] Lehmann H. E. Kline N. S. (1983). Clinical discoveries with antidepressant drugs, in Discoveries in Pharmacology, eds Parnham M. J. Bruinvels J. (Amsterdam: Elsevier), 209–221.

[B71] Levitt P. Pintar J. E. Breakefield X. O. (1982). Immunocytochemical demonstration of monoamine oxidase B in brain astrocytes and serotonergic neurons. Proc. Natl. Acad. Sci. U.S.A. 79, 6385–6389. 10.1073/pnas.79.20.6385 6755469 PMC347126

[B72] Lotufo-Neto F. Trivedi M. Thase M. E. (1999). Meta-analysis of the reversible inhibitors of monoamine oxidase type A moclobemide and brofaromine for the treatment of depression. Neuropsychopharmacology 20, 226–247. 10.1016/S0893-133X(98)00075-X 10063483

[B73] Magyar K. (2011). The pharmacology of selegiline. Int. Rev. Neurobiol. 100, 65–84. 10.1016/B978-0-12-386467-3.00004-2 21971003

[B74] Mann J. J. Aarons S. F. Wilner P. J. Keilp J. G. Sweeney J. A. Pearlstein T.. (1989). A controlled study of the antidepressant efficacy and side effects of (−)-deprenyl. A selective monoamine oxidase inhibitor. Arch. Gen. Psychiatry 46, 45–50. 10.1001/archpsyc.1989.01810010047007 2491941

[B75] Marek K. L. Friedman J. Hauser R. Juncos J. LeWitt P. Mijawaki E. (1997). Phase II evaluation of rasagiline mesylate (TVP-1012), a novel anti-parkinsonian drug, in parkinsonian patients not receiving levodopa/carbidopa. Mov. Disord. 12, 838.

[B76] Maruyama W. Akao Y. Youdim M. B. Davis B. A. Naoi M. (2001). Transfection-enforced Bcl-2 overexpression and an anti-Parkinson drug, rasagiline, prevent nuclear accumulation of glyceraldehyde-3-phosphate dehydrogenase induced by an endogenous dopaminergic neurotoxin, N-methyl(R)salsolinol. J. Neurochem. 78, 727–735. 10.1046/j.1471-4159.2001.00448.x 11520893

[B77] Maruyama W. Weinstock M. Youdim M. B. Nagai M. Naoi M. (2003). Anti-apoptotic action of anti-Alzheimer drug, TV3326 [(N-propargyl)-(3R)-aminoindan-5-yl]-ethyl methyl carbamate, a novel cholinesterase-monoamine oxidase inhibitor. Neurosci. Lett. 341, 233–236. 10.1016/S0304-3940(03)00211-8 12697291

[B78] Mawhinney M. Cole D. Azzaro A. J. (2003). Daily transdermal administration of selegiline to guinea-pigs preferentially inhibits monoamine oxidase activity in brain when compared with intestinal and hepatic tissues. J. Pharm. Pharmacol. 55, 27–34. 10.1111/j.2042-7158.2003.tb02430.x 12625864

[B79] Minders C. Petzer J. P. Petzer A. Lourens A. C. (2015). Monoamine oxidase inhibitory activities of heterocyclic chalcones. Bioorg. Med. Chem. Lett. 25, 5270–5276. 10.1016/j.bmcl.2015.09.049 26432037

[B80] Mohammadi M. R. Ghanizadeh A. Alaghband-Rad J. Tehranidoost M. Mesgarpour B. Soori H. (2004). Selegiline in comparison with methylphenidate in attention deficit hyperactivity disorder children and adolescents in a double-blind, randomized clinical trial. J. Child Adolesc. Psychopharmacol. 14, 418–425. 10.1089/cap.2004.14.418 15650498

[B81] Molochnikov L. Rabey J. M. Dobronevsky E. Bonucelli U. Ceravolo R. Frosini D.. (2012). A molecular signature in blood identifies early Parkinson's disease. Mol. Neurodegener. 7:26. 10.1186/1750-1326-7-26 22651796 PMC3424147

[B82] Moradov D. Finkin-Groner E. Bejar C. Sunita P. Schorer-Apelbaum D. Barasch D.. (2015). Dose-limiting inhibition of acetylcholinesterase by ladostigil results from the rapid formation and fast hydrolysis of the drug-enzyme complex formed by its major metabolite, R-MCPAI. Biochem. Pharmacol. 94, 164–172. 10.1016/j.bcp.2015.01.017 25662585

[B83] Myllyla V. V. Sotaniemi K. A. Hakulinen P. Maki-Ikola O. Heinonen E. H. (1997). Selegiline as the primary treatment of Parkinson's disease–a long-term double-blind study. Acta Neurol. Scand. 95, 211–218. 10.1111/j.1600-0404.1997.tb00101.x 9150811

[B84] Myllyla V. V. Sotaniemi K. A. Vuorinen J. A. Heinonen E. H. (1992). Selegiline as initial treatment in *de novo* parkinsonian patients. Neurology 42, 339–343. 10.1212/WNL.42.2.339 1736162

[B85] Naylor G. J. Smith A. H. Connelly P. (1987). A controlled trial of methylene blue in severe depressive illness. Biol. Psychiatry 22, 657–659. 10.1016/0006-3223(87)90194-6 3555627

[B86] Niederhofer H. (2003). Selegiline and methylphenidate in treatment of ADHD. Psychiatr. Danub. 15, 3–6. 19112366

[B87] Olanow C. W. Myllyla V. V. Sotaniemi K. A. Larsen J. P. Palhagen S. Przuntek H.. (1998). Effect of selegiline on mortality in patients with Parkinson's disease: a meta-analysis. Neurology 51, 825–830. 10.1212/WNL.51.3.825 9748034

[B88] Olanow C. W. Rascol O. Hauser R. Feigin P. D. Jankovic J. Lang A.. (2009). A double-blind, delayed-start trial of rasagiline in Parkinson's disease. N. Engl. J. Med. 361, 1268–1278. 10.1056/NEJMoa0809335 19776408

[B89] Palhagen S. Heinonen E. Hagglund J. Kaugesaar T. Maki-Ikola O. Palm R. (2006). Selegiline slows the progression of the symptoms of Parkinson disease. Neurology 66, 1200–1206. 10.1212/01.wnl.0000204007.46190.54 16540603

[B90] Perez-Lloret S. Rey M. V. Montastruc J. L. Rascol O. (2013). Adverse drug reactions with selegiline and rasagiline compared to levodopa and ropinirole: a study in the French Pharmacovigilance Database. J. Neurol. Sci. 333, e129 10.1016/j.jns.2013.07.432

[B91] Parkinson Study Group (1989). Effect of deprenyl on the progression of disability in early Parkinson's disease. N. Engl. J. Med. 321, 1364–1371. 10.1056/NEJM198911163212004 2509910

[B92] Parkinson Study Group (1993). Effects of tocopherol and deprenyl on the progression of disability in early Parkinson's disease. N. Engl. J. Med. 328, 176–183. 10.1056/NEJM199301213280305 8417384

[B93] Parkinson Study Group (1996a). Impact of deprenyl and tocopherol treatment on Parkinson's disease in DATATOP patients requiring levodopa. Ann. Neurol. 39, 37–45. 10.1002/ana.410390107 8572664

[B94] Parkinson Study Group (1996b). Impact of deprenyl and tocopherol treatment on Parkinson's disease in DATATOP subjects not requiring levodopa. Ann. Neurol. 39, 29–36. 8572663 10.1002/ana.410390106

[B95] Parkinson Study Group (2002). A controlled trial of rasagiline in early Parkinson disease: the TEMPO Study. Arch. Neurol. 59, 1937–1943. 10.1001/archneur.59.12.1937 12470183

[B96] Parkinson Study Group (2004). A controlled, randomized, delayed-start study of rasagiline in early Parkinson disease. Arch. Neurol. 61, 561–566. 10.1001/archneur.61.4.561 15096406

[B97] Przuntek H. Conrad B. Dichgans J. Kraus P. H. Krauseneck P. Pergande G.. (1999). SELEDO: a 5-year long-term trial on the effect of selegiline in early Parkinsonian patients treated with levodopa. Eur. J. Neurol. 6, 141–150. 10.1111/j.1468-1331.1999.tb00007.x 10053226

[B98] Rabey J. M. Sagi I. Huberman M. Melamed E. Korczyn A. Giladi N.. (2000). Rasagiline mesylate, a new MAO-B inhibitor for the treatment of Parkinson's disease: a double-blind study as adjunctive therapy to levodopa. Clin. Neuropharmacol. 23, 324–330. 10.1097/00002826-200011000-00005 11575866

[B99] Ramsay R. R. Dunford C. Gillman P. K. (2007). Methylene blue and serotonin toxicity: inhibition of monoamine oxidase A (MAO A) confirms a theoretical prediction. Br. J. Pharmacol. 152, 946–951. 10.1038/sj.bjp.0707430 17721552 PMC2078225

[B100] Rascol O. Brooks D. J. Melamed E. Oertel W. Poewe W. Stocchi F.. (2005). Rasagiline as an adjunct to levodopa in patients with Parkinson's disease and motor fluctuations (LARGO, Lasting effect in Adjunct therapy with Rasagiline Given Once daily, study): a randomised, double-blind, parallel-group trial. Lancet 365, 947–954. 10.1016/S0140-6736(05)71083-7 15766996

[B101] Rascol O. Fitzer-Attas C. J. Hauser R. Jankovic J. Lang A. Langston J. W.. (2011). A double-blind, delayed-start trial of rasagiline in Parkinson's disease (the ADAGIO study): prespecified and *post-hoc* analyses of the need for additional therapies, changes in UPDRS scores, and non-motor outcomes. Lancet Neurol. 10, 415–423. 10.1016/S1474-4422(11)70073-4 21482191

[B102] Reichmann H. Sommer U. Fuchs G. Hefter H. Mark G. Muller T.. (2000). Workshop IV: drug treatment guidelines for the long-term management of Parkinson's disease. J. Neurol. 247(Suppl. 4), IV/40–41. 10.1007/PL00007776 11199819

[B103] Reynolds G. P. Elsworth J. D. Blau K. Sandler M. Lees A. J. Stern G. M. (1978). Deprenyl is metabolized to methamphetamine and amphetamine in man. Br. J. Clin. Pharmacol. 6, 542–544. 10.1111/j.1365-2125.1978.tb00883 728327 PMC1429688

[B104] Riederer P. Lachenmayer L. Laux G. (2004). Clinical applications of MAO-inhibitors. Curr. Med. Chem. 11, 2033–2043. 10.2174/0929867043364775 15279566

[B105] Riederer P. Laux G. (2011). MAO-inhibitors in Parkinson's Disease. Exp. Neurobiol. 20, 1–17. 10.5607/en.2011.20.1.1 22110357 PMC3213739

[B106] Rubinstein S. Malone M. A. Roberts W. Logan W. J. (2006). Placebo-controlled study examining effects of selegiline in children with attention-deficit/hyperactivity disorder. J. Child Adolesc. Psychopharmacol. 16, 404–415. 10.1089/cap.2006.16.404 16958566

[B107] Sader-Mazbar O. Loboda Y. Finberg J. P. M. (2013). Increased L-dopa-derived dopamine following selective MAO-A or -B inhibition in rat striatum depleted of dopaminergic and serotonergic innervation. Br. J. Pharmacol. 170, 999–1013. 10.1111/bph.12349 23992249 PMC3949649

[B108] Sandoval-Rincon M. Saenz-Farret M. Miguel-Puga A. Micheli F. Arias-Carrion O. (2015). Rational pharmacological approaches for cognitive dysfunction and depression in Parkinson's disease. Front. Neurol. 6:71. 10.3389/fneur.2015.00071 25873910 PMC4379942

[B109] Schapira A. Bate G. Kirkpatrick P. (2005). Rasagiline. Nat. Rev. Drug Discov. 4, 625–626. 10.1038/nrd1803 16106586

[B110] Schapira A. H. Stocchi F. Borgohain R. Onofrj M. Bhatt M. Lorenzana P.. (2013). Long-term efficacy and safety of safinamide as add-on therapy in early Parkinson's disease. Eur. J. Neurol. 20, 271–280. 10.1111/j.1468-1331.2012.03840.x 22967035

[B111] Shoham S. Bejar C. Kovalev E. Schorer-Apelbaum D. Weinstock M. (2007). Ladostigil prevents gliosis, oxidative-nitrative stress and memory deficits induced by intracerebroventricular injection of streptozotocin in rats. Neuropharmacology 52, 836–843. 10.1016/j.neuropharm.2006.10.005 17123555

[B112] Shoulson I. Oakes D. Fahn S. Lang A. Langston J. W. LeWitt P.. (2002). Impact of sustained deprenyl (selegiline) in levodopa-treated Parkinson's disease: a randomized placebo-controlled extension of the deprenyl and tocopherol antioxidative therapy of parkinsonism trial. Ann. Neurol. 51, 604–612. 10.1002/ana.10191 12112107

[B113] Shulman K. I. Herrmann N. Walker S. E. (2013). Current place of monoamine oxidase inhibitors in the treatment of depression. CNS Drugs 27, 789–797. 10.1007/s40263-013-0097-3 23934742

[B114] Son S. Y. Ma J. Kondou Y. Yoshimura M. Yamashita E. Tsukihara T. (2008). Structure of human monoamine oxidase A at 2.2-A resolution: the control of opening the entry for substrates/inhibitors. Proc. Natl. Acad. Sci. U.S.A. 105, 5739–5744. 10.1073/pnas.0710626105 18391214 PMC2311356

[B115] Stocchi F. Arnold G. Onofrj M. Kwiecinski H. Szczudlik A. Thomas A.. (2004). Improvement of motor function in early Parkinson disease by safinamide. Neurology 63, 746–748. 10.1212/01.WNL.0000134672.44217.F7 15326260

[B116] Stocchi F. Borgohain R. Onofrj M. Schapira A. H. Bhatt M. Lucini V.. (2012). A randomized, double-blind, placebo-controlled trial of safinamide as add-on therapy in early Parkinson's disease patients. Mov. Disord. 27, 106–112. 10.1002/mds.23954 21913224

[B117] Stocchi F. Rabey J. M. (2011). Effect of rasagiline as adjunct therapy to levodopa on severity of OFF in Parkinson's disease. Eur. J. Neurol. 18, 1373–1378. 10.1111/j.1468-1331.2011.03512.x 21895884

[B118] Stocchi F. Vacca L. Grassini P. De Pandis M. F. Battaglia G. Cattaneo C.. (2006). Symptom relief in Parkinson disease by safinamide: biochemical and clinical evidence of efficacy beyond MAO-B inhibition. Neurology 67, S24–S29. 10.1212/WNL.67.7_suppl_2.S24 17030737

[B119] Sunderland T. Cohen R. M. Molchan S. Lawlor B. A. Mellow A. M. Newhouse P. A.. (1994). High-dose selegiline in treatment-resistant older depressive patients. Arch. Gen. Psychiatry 51, 607–615. 10.1001/archpsyc.1994.03950080019003 7519005

[B120] Tatton W. G. Chalmers-Redman R. M. E. (1996). Modulation of gene expression rather than monoamine oxidase inhibition: (−)-Deprenyl-related compounds in controlling neurodegeneration. Neurology 47, S171–S183. 10.1212/WNL.47.6_Suppl_3.171S 8959986

[B121] Tetrud J. W. Langston J. W. (1989). The effect of deprenyl (selegiline) on the natural history of Parkinson's disease. Science 245, 519–522. 10.1126/science.2502843 2502843

[B122] Thebault J. J. Guillaume M. Levy R. (2004). Tolerability, safety, pharmacodynamics, and pharmacokinetics of rasagiline: a potent, selective, and irreversible monoamine oxidase type B inhibitor. Pharmacotherapy 24, 1295–1305. 10.1592/phco.24.14.1295.43156 15628826

[B123] Tipton K. F. Dostert P. Strolin Benedetti M. (eds.). (1984). Monoamine Oxidase and Disease. London: Academic Press.

[B124] Todd K. G. Baker G. B. (2008). Neurochemical effects of the monoamine oxidase inhibitor phenelzine on brain GABA and alanine: a comparison with vigabatrin. J. Pharm. Pharm. Sci. 11, 14s–21s. 10.18433/J34S38 19203467

[B125] Waldmeier P. C. Boulton A. A. Cools A. R. Kato A. C. Tatton W. G. (2000). Neurorescuing effects of the GAPDH ligand CGP B. J. Neural. Transm. Suppl. 60, 197–214. 11205140 10.1007/978-3-7091-6301-6_13

[B126] Wang L. Esteban G. Ojima M. Bautista-Aguilera O. M. Inokuchi T. Moraleda I.. (2014). Donepezil + propargylamine + 8-hydroxyquinoline hybrids as new multifunctional metal-chelators, ChE and MAO inhibitors for the potential treatment of Alzheimer's disease. Eur. J. Med. Chem. 80, 543–561. 10.1016/j.ejmech.2014.04.078 24813882

[B127] Waters C. H. Sethi K. D. Hauser R. A. Molho E. Bertoni J. M. (2004). Zydis selegiline reduces off time in Parkinson's disease patients with motor fluctuations: a 3-month, randomized, placebo-controlled study. Mov. Disord. 19, 426–432. 10.1002/mds.20036 15077240

[B128] Weinreb O. Amit T. Bar-Am O. Chillag-Talmor O. Youdim M. B. (2005). Novel neuroprotective mechanism of action of rasagiline is associated with its propargyl moiety: interaction of Bcl-2 family members with PKC pathway. Ann. N. Y. Acad. Sci. 1053, 348–355. 10.1196/annals.1344.030 16179541

[B129] Weinreb O. Amit T. Bar-Am O. Youdim M. B. (2012). Ladostigil: a novel multimodal neuroprotective drug with cholinesterase and brain-selective monoamine oxidase inhibitory activities for Alzheimer's disease treatment. Curr. Drug Targets 13, 483–494. 10.2174/138945012799499794 22280345

[B130] Weinreb O. Amit T. Riederer P. Youdim M. B. Mandel S. A. (2011). Neuroprotective profile of the multitarget drug rasagiline in Parkinson's disease. Int. Rev. Neurobiol. 100, 127–149. 10.1016/B978-0-12-386467-3.00007-8 21971006

[B131] Weinreb O. Badinter F. Amit T. Bar-Am O. Youdim M. B. (2015). Effect of long-term treatment with rasagiline on cognitive deficits and related molecular cascades in aged mice. Neurobiol. Aging 36, 2628–2636. 10.1016/j.neurobiolaging.2015.05.009 26142126

[B132] Weinstock M. Bejar C. Wang R. H. Poltyrev T. Gross A. Finberg J. P.. (2000). TV3326, a novel neuroprotective drug with cholinesterase and monoamine oxidase inhibitory activities for the treatment of Alzheimer's disease. J. Neural. Transm. Suppl. 60, 157–169. 10.1007/978-3-7091-6301-6_10 11205137

[B133] Westfall T. C. Westfall D. P. (2011). Neurotransmission: the autonomic and somatic motor nervous systems, in The Pharmacological Basis of Therapeutics, 12th Edn, ed Brunton L. L. (New York, NY: McGraw Hill), 171–218.

[B134] Westlund K. N. Denney R. M. Rose R. M. Abell C. W. (1988). Localization of distinct monoamine oxidase A and monoamine oxidase B cell populations in human brainstem. Neuroscience 25, 439–456. 10.1016/0306-4522(88)90250-3 3399053

[B135] Westlund K. N. Krakower T. J. Kwan S. W. Abell C. W. (1993). Intracellular distribution of monoamine oxidase A in selected regions of rat and monkey brain and spinal cord. Brain Res. 612, 221–230. 10.1016/0006-8993(93)91664-E 8330200

[B136] Winger G. D. Yasar S. Negus S. S. Goldberg S. R. (1994). Intravenous self-administration studies with l-deprenyl (selegiline) in monkeys. Clin. Pharmacol. Ther. 56, 774–780. 10.1038/clpt.1994.208 7995020

[B137] Yasar S. Goldberg J. P. Goldberg S. R. (1996). Are metabolites of l-deprenyl (selegiline) useful or harmful? Indications from preclinical research. J. Neural. Transm. Suppl. 48, 61–73. 10.1007/978-3-7091-7494-4_6 8988462

[B138] Yogev-Falach M. Amit T. Bar-Am O. Weinstock M. Youdim M. B. (2002). Involvement of MAP kinase in the regulation of amyloid precursor protein processing by novel cholinesterase inhibitors derived from rasagiline. FASEB J. 16, 1674–1676. 10.1096/fj.02-0198fje 12206996

[B139] Youdim M. B. Finberg J. P. (1983). Monoamine oxidase inhibitor antidepressants, in Part 1: Preclinical Psychopharmacology, eds Grahame-Smith D. G. Cowen P. J. (Amsterdam: Excerpta Medica), 38–70.

[B140] Youdim M. B. Fridkin M. Zheng H. (2005). Bifunctional drug derivatives of MAO-B inhibitor rasagiline and iron chelator VK-28 as a more effective approach to treatment of brain ageing and ageing neurodegenerative diseases. Mech. Ageing Dev. 126, 317–326. 10.1016/j.mad.2004.08.023 15621213

[B141] Yu P. H. Hertz L. (1983). Type A and B monoamine oxidase in glial cells in long-term culture. Prog. Neuropsychopharmacol. Biol. Psychiatry 7, 687–690. 10.1016/0278-5846(83)90046-5 6686700

[B142] Zheng H. Gal S. Weiner L. M. Bar-Am O. Warshawsky A. Fridkin M.. (2005). Novel multifunctional neuroprotective iron chelator-monoamine oxidase inhibitor drugs for neurodegenerative diseases: *in vitro* studies on antioxidant activity, prevention of lipid peroxide formation and monoamine oxidase inhibition. J. Neurochem. 95, 68–78. 10.1111/j.1471-4159.2005.03340.x 16181413

